# Do we have an “anti-stigmotic”? – Addressing Mental-Illness Related Stigma as the main issue

**DOI:** 10.1192/j.eurpsy.2023.2187

**Published:** 2023-07-19

**Authors:** C. Cabaços, J. Andrade, F. Pocinho, M. Carneiro, G. Santos, D. Loureiro, A. Macedo

**Affiliations:** 1 Institute of Psychological Medicine, Faculty of Medicine, University of Coimbra; 2 Psychiatry Department, Centro Hospitalar e Universitário de Coimbra, Coimbra, Portugal

## Abstract

**Introduction:**

For people with mental illness, internalized stigma, also referred to as self-stigma, is characterized by a subjective perception of devaluation, marginalization, secrecy, shame, and withdrawal. It has many adverse effects on individual’s psychological well-being and clinical outcomes. The iatrogenic effects it has during psychotherapeutic treatment can significantly reduce utilization of mental health care services, reduce quality of life and increase avoidant coping. Overall, internalized stigma is considered a risk factor for poorer mental health prognosis. Although some interventions have recently been developed to specifically intervene on this target as part of psychological recovery goals over the course of treatment, most clinicians are not yet aware or empowered to correctly address this.

**Objectives:**

Description of a clinical case illustrating the relevance on addressing internalized mental illness related stigma during the recovery process.

**Methods:**

Clinical case report and review of the literature on the subject.

**Results:**

We present the case of a 47-year-old female patient, C.S., single, graduated in social work (currently unemployed), who was admitted at the Psychiatry Day Hospital, where she was referred by her Psychiatry Assistant because of abulia, social withdrawal and isolation, depressed mood, thoughts of shame, guilt and self-devaluation and work incapacity. She had been admitted in the Psychiatry ward one year earlier for a first psychotic breakthrough, presenting persecutory and grandiose delusions and auditory hallucinations. After three weeks of inpatient treatment with antipsychotics, a full remission of the symptoms was achieved, without any posterior relapse. Before that first psychotic episode, the patient had been taking anti-depressive medication (escitalopram 20 mg id) for many years, prescribed by her General Practitioner, for mild to moderate depressive symptoms. After being discharged from the Psychiatry ward, C. kept following an outpatient treatment with anti-depressives and behavioural activation-based psychotherapy. She started to believe she was mentally ill and therefore weak, uncapable, and less deserving than her peers or her previous self. These self-stigmatizing ideas were enhanced by the lack of family support and the beliefs that were fostered by her mother, with whom she started to live after the hospitalization. These factors led to a dysfunctional internalization of an illness behaviour, jeopardizing the patient’s ability to reach full recovery.

**Conclusions:**

This case reinforces the importance of targeting mental illness related stigma during the recovery process. Also, involving the family is of extreme importance to achieve support and address shared beliefs and the interchange between social and internalized stigma.

**Disclosure of Interest:**

None Declared

